# Generation and transcriptomic characterization of *MIR137* knockout miniature pig model for neurodevelopmental disorders

**DOI:** 10.1186/s13578-024-01268-8

**Published:** 2024-06-28

**Authors:** Shengyun Xu, Jiaoxiang Wang, Kexin Mao, Deling Jiao, Zhu Li, Heng Zhao, Yifei Sun, Jin Feng, Yuanhao Lai, Ruiqi Peng, Yu Fu, Ruoyi Gan, Shuhan Chen, Hong-Ye Zhao, Hong-Jiang Wei, Ying Cheng

**Affiliations:** 1 Institute of Biomedical Research, Yunnan University, Kunming, 650500 China; 2 Key Laboratory for Porcine Gene Editing and Xenotransplantation in Yunnan Province, Yunnan Agricultural University, Kunming, 650201 China; 3 Southwest United Graduate School, Kunming, 650092 China

**Keywords:** Pig, Neurodevelopmental disorder, miR-137, Autism spectrum disorders, Intellectual disorders, Animal model

## Abstract

**Background:**

Neurodevelopmental disorders (NDD), such as autism spectrum disorders (ASD) and intellectual disorders (ID), are highly debilitating childhood psychiatric conditions. Genetic factors are recognized as playing a major role in NDD, with a multitude of genes and genomic regions implicated. While the functional validation of NDD-associated genes has predominantly been carried out using mouse models, the significant differences in brain structure and gene function between mice and humans have limited the effectiveness of mouse models in exploring the underlying mechanisms of NDD. Therefore, it is important to establish alternative animal models that are more evolutionarily aligned with humans.

**Results:**

In this study, we employed CRISPR/Cas9 and somatic cell nuclear transplantation technologies to successfully generate a knockout miniature pig model of the *MIR137* gene, which encodes the neuropsychiatric disorder-associated microRNA miR-137. The homozygous knockout of *MIR137* (*MIR137*^*–/–*^) effectively suppressed the expression of mature miR-137 and led to the birth of stillborn or short-lived piglets. Transcriptomic analysis revealed significant changes in genes associated with neurodevelopment and synaptic signaling in the brains of *MIR137*^*–/–*^ miniature pig, mirroring findings from human ASD transcriptomic data. In comparison to miR-137-deficient mouse and human induced pluripotent stem cell (hiPSC)-derived neuron models, the miniature pig model exhibited more consistent changes in critical neuronal genes relevant to humans following the loss of miR-137. Furthermore, a comparative analysis identified differentially expressed genes associated with ASD and ID risk genes in both miniature pig and hiPSC-derived neurons. Notably, human-specific miR-137 targets, such as *CAMK2A*, known to be linked to cognitive impairments and NDD, exhibited dysregulation in *MIR137*^*–/–*^ miniature pigs. These findings suggest that the loss of miR-137 in miniature pigs affects genes crucial for neurodevelopment, potentially contributing to the development of NDD.

**Conclusions:**

Our study highlights the impact of miR-137 loss on critical genes involved in neurodevelopment and related disorders in *MIR137*^*–/–*^ miniature pigs. It establishes the miniature pig model as a valuable tool for investigating neurodevelopmental disorders, providing valuable insights for potential applications in human research.

**Supplementary Information:**

The online version contains supplementary material available at 10.1186/s13578-024-01268-8.

## Introduction

Neurodevelopmental disorders (NDD) are the most disabling childhood psychiatric disorders, believed to result from the combined effects of multiple genetic and environmental factors, yet the specific pathogenic mechanisms remain largely unclear [1, 2]. NDD primarily include autism spectrum disorders (ASD), intellectual disorders (ID), attention deficit hyperactivity disorder (ADHD) and manifest as deficits in intelligence, cognition, mental status, motor skills, and social behavior, often accompanied by epileptic symptoms [3]. Genetic factors play a predominant role in the pathogenesis of NDD [4]. For instance, the 5-Contry Cohort study suggested that the heritability of ASD was around 80% [5], and meta-analyses of twin studies estimated it to be in the range of 64–91% [6]. Being one of the most heritable common disorders, current research has identified over 100 genes and genomic regions associated with ASD [7, 8]. These genetic alterations can result from single nucleotide changes or variations in DNA fragments, known as copy number variations [9, 10]. A 2018 study in the United States revealed a rising prevalence rate (overall 2.67%) of ASD among 8-year-olds, with ASD being 3.8 times more common in boys than girls [11]. In China, a cohort study involving approximately 120,000 participants found the prevalence of ASD among children aged 6 to 12 years to be around 0.70%, with a 3.2 times higher prevalence in boys than girls [12]. Despite these findings, the specific pathogenesis of most ASD cases remains largely unclear due to significant heterogeneity.

MicroRNAs (miRNAs) are a class of approximately 22-nucleotide (nt) non-coding RNAs that, through post-transcriptional regulation, negatively regulate mRNA stability or translation, thereby altering various cellular functions [13]. Numerous studies have directly linked miRNA dysregulation to neurodevelopment and various neuropsychiatric disorders [14]. For example, the neuronal deficiency of endoribonuclease DICER, a core component involved in miRNA biogenesis, can lead to neuronal differentiation defects and abnormal brain structures [15]. Dysregulation of specific miRNAs, including miR-124, miR-137, miR-128, miR-218, has been shown to influence synaptic growth, neuronal plasticity, electrophysiology, and behavior in mouse models [16–19]. Among these miRNAs, miR-137, encoded by the *MIR137* gene, is highly conserved in both mammalian and non-mammalian vertebrates [20], and strongly correlated with development, differentiation, and maturation of the nervous system [21]. Multiple human genome-wide association studies (GWAS) have revealed a strong genetic association between *MIR137* and neuropsychiatric disorders, particularly schizophrenia, ASD and ID [22–26]. Genetic experiments have shown that overexpression of miR-137 is linked to schizophrenia [27, 28]. Conversely, clinical case reports have confirmed that patients with *MIR137* heterozygous microdeletion (1p21.3 microdeletion) are all diagnosed with ID, and 90% of them exhibit clinical symptoms associated with ASD [29–31]. In our previous studies using mouse models, we observed that complete loss of miR-137, either in the germline or in specific brain cell types, resulted in postnatal lethality [17, 32]. Additionally, partial loss of miR-137 was associated with impaired behavioral and cellular phenotypes linked to ASD [17].

Given the significant differences in brain structure and gene functionality between rodent models and humans, there is an urgent need to establish new animal models for NDD using species that are evolutionarily closer to humans, such as pigs [33]. Pigs offer advantages in anatomy, physiology, nutritional metabolism, and ethical considerations when compared to rodents [34]. They can carry mutations with similar pathogenicity to humans, making them an ideal model for studying human diseases [35–37]. In biomedicine, gene editing and somatic cell cloning technologies have been used to generate diverse pig models for human diseases, including diabetes, cardiovascular disease, genetic disorders, tumors, and neurodegenerative diseases [38–41]. The multigyral structure of pig brains more closely resembles that of humans, exhibiting cortical folds and nerve fiber connectivity more effectively than rodent brains [42]. Similar to humans, pigs have a comparable white matter composition, suggesting similarities in brain injury and recovery mechanisms [43]. Promising task types for studying social novelty and cognition have been identified in pig models [44–46]. Moreover, the larger litter size of pigs also makes them an ideal model for drug screening [40].

In this study, we utilized CRISPR/Cas9 in conjunction with somatic cell nuclear transplantation (SCNT) to generate a *MIR137* knockout (KO, *MIR137*^*–/–*^) miniature pig model. Through transcriptomics analysis, we identified a significant number of genes associated with brain development and NDD that were dysregulated upon loss of miR-137. By comparing our results with RNA-seq data from miR-137-deficient human induced pluripotent stem cells (hiPSC)-derived forebrain neurons and fetal mouse forebrain, we demonstrated that the miniature pig exhibited more similar transcriptomic features reflecting changes in humans. Additionally, we discovered human-specific miR-137 target genes that were altered in the brain tissue of *MIR137*^*–/–*^ miniature pig. Overall, our miniature pig model shows promise as an ideal animal model for NDD and provides valuable insight for utilizing miniature pigs in the study of neurodevelopment and NDD.

## Results

### Generation of ***MIR137***^***–/–***^ miniature pig model

**Fig. 1:**
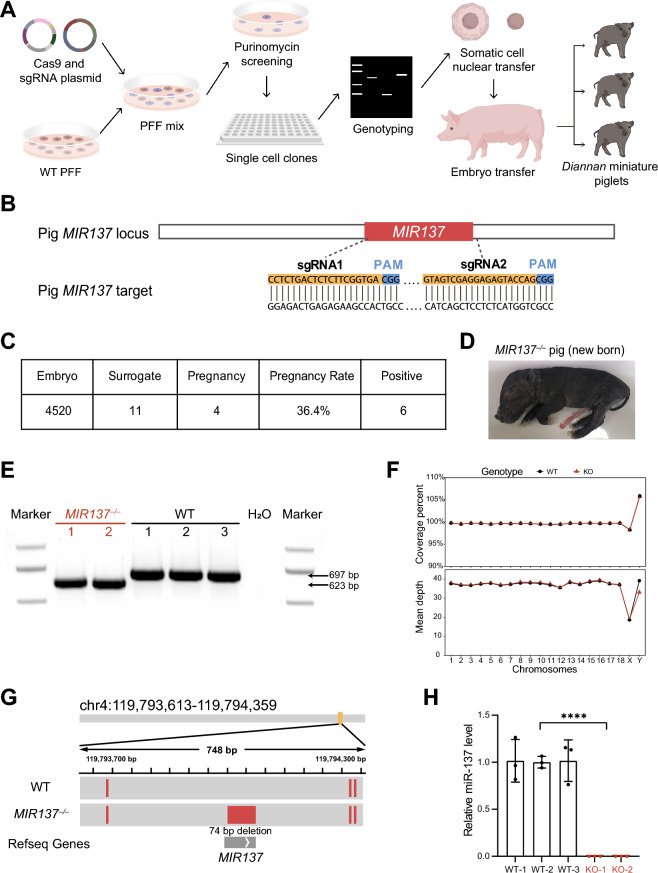
Generation of *MIR137* knockout (*MIR137*^*–/–*^) miniature pigs. **A** Experimental procedures for *MIR137*^*–/–*^ miniature pig model generation. PFF, porcine fetal fibroblast. **B** Schematic diagram of sgRNA-targeting sites in the porcine *MIR137* gene. **C** Summary of embryo transfer data from somatic cell nuclear transplantation (SCNT) of the *MIR137*^*–/–*^ embryo to generate mutant miniature pig models. **D** Representative image of a newborn *MIR137*^*–/–*^ piglet. **E** PCR genotyping results of the *MIR137*^*–/–*^ miniature pigs. **F** Genomic coverage of PCR-free whole-genome sequencing (WGS). **G** PCR-free WGS analysis revealed a 74-bp deletion of *MIR137* in the genome. **H** Quantitative RT-PCR analysis of mature miR-137 expression levels in *MIR137*^*–/–*^ miniature pigs and wild-type (WT) control miniature pigs. Data are represented as mean ± SEM. Statistical significance was tested with unpaired two-tailed t tests, with ****p < 0.0001

To investigate the roles of miR-137 in miniature pig models, we utilized a local pig strain called *Diannan* small-eared (DSE) miniature pigs. The method employed to generate the *MIR137* knockout (KO, *MIR137*^*–/–*^) miniature pig model is illustrated in Fig. 1A and described previously by Shen et al. [47]. Briefly, sgRNAs targeting the pig *MIR137* gene were designed, and the sgRNAs with the highest cutting efficiency (sgRNA1, 17%; and sgRNA2, 32%) were selected to construct PGL3-U6-sgRNA-Puro plasmids (Fig. 1B, Supplementary Fig. 1A–C and Supplementary Table 1). After transfecting PGL3-U6-sgRNA-Puro and Cas9 plasmids into porcine fetal fibroblasts, we obtained several single-cell colonies. Subsequent genotyping of the cell colonies yielded two colonies with deletion of 73-bp (C3) and 74-bp (C4), respectively, which served as donor cells for SCNT (Supplementary Fig. 1D, E). These reconstructed embryos were then transplanted into 11 recipient gilts, resulting in pregnancy in four of them and the birth of 6 piglets (Fig. 1C, D). Polymerase chain reaction (PCR) was utilized to confirm the genotype of the *MIR137* KO miniature pigs, and all 6 piglets were identified as homozygous (*MIR137*^*–/–*^) (Fig. 1E and Supplementary Fig. 1D, E). Unfortunately, all *MIR137*^*–/–*^ piglets were stillborn or died shortly after birth, preventing the acquisition of any young or adult *MIR137*^*–/–*^ miniature pigs.

To further validate the deletion efficiency of CRISPR/Cas9-based gene editing, we performed PCR-free whole genome sequencing (WGS) using DNA obtained from a *MIR137*^*–/–*^ miniature pig (originated from C4) and a wild-type (WT) control miniature pig. The WGS data covers approximately 40 × sequencing depth, enabling the detection of insertions and deletions (InDels) in the whole genome (Fig. 1F). Our analysis clearly indicated a 74-bp deletion within the *MIR137* gene (Fig. 1G). Additionally, quantitative RT-PCR analysis demonstrated that the expression level of mature miR-137 nearly abolished in *MIR137*^*–/–*^ miniature pigs compared to WT control miniature pigs (Fig. 1H). These results confirmed the specificity and efficiency of knocking out the *MIR137* gene in our miniature pig models.

### Depletion of *MIR137* induces significant alterations in gene expression in the brain of miniature pigs

**Fig. 2:**
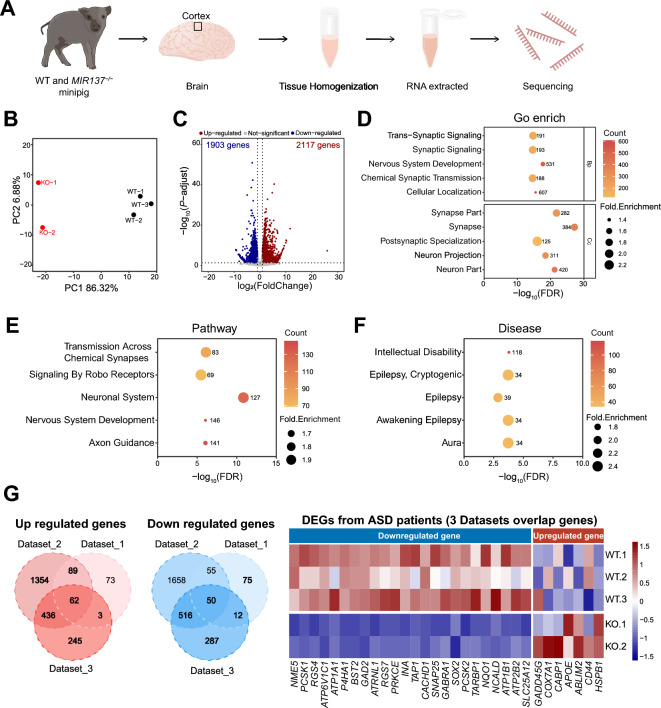
*MIR137* deletion leads to a distinct transcriptomic change in miniature pig brain. **A** Experiment flow for RNA-seq. **B** Principal component analysis of WT (n = 3) and *MIR137*^*–/–*^ (n = 2) miniature pigs. **C** Volcano plot illustrating differentially expressed genes (DEGs) in WT and *MIR137*^*–/–*^ miniature pigs. A total of 2,117 upregulated and 1,903 downregulated DEGs were identified in the *MIR137*^*–/–*^ miniature pig brain (cutoff thresholds: log_2_ fold change > 1 or < −1, and adjusted p < 0.05). **D**–**F** Top 5 GO terms (**D**), enriched pathways (**E**) and disease associations (**F**) for DEGs identified in the *MIR137*^*–/–*^ miniature pig brain. **G** Heatmap showing gene expression for the overlapped DEGs identified in the *MIR137*^*–/–*^ miniature pig brain with the DEGs identified in ASD patient brain from three different datasets. The complete gene list is provided in Supplementary Table 3

To explore the transcriptomic changes upon miR-137, we used RNA sequencing (RNA-seq) to compare cerebral cortex obtained from two *MIR137*^*–/–*^ and three newborn WT control miniature pig (Fig. 2A). Principal component analysis (PCA) revealed a high degree of similarity among the biological replicates and a clear distinction between *MIR137*^*–/–*^ and WT miniature pigs (Fig. 2B). Our RNA-seq analysis highlighted a significant alteration in gene expression due to the loss of miR-137. In total, we identified 2,117 upregulated differentially expressed genes (DEGs) and 1,903 downregulated DEGs in *MIR137*^*–/–*^ miniature pigs compared to WT miniature pigs (Fig. 2C and Supplementary Table 2).

Gene Ontology (GO) analysis revealed that these DEGs were significantly enriched in biological processes related to nervous system development, cellular localization and synaptic signaling. They were also found to be enriched in functional genes involved in neuron and synapse formation (Fig. 2D). Pathway enrichment analysis indicated that the DEGs are significantly involved in neuronal system development, transmission across chemical synapses, and axon guidance (Fig. 2E). Interestingly, we observed a strong association between these DEGs and several human neurological diseases, including ID, aura and epilepsy (Fig. 2F). We then proceeded to compare the DEGs of *MIR137*^*–/–*^ miniature pigs with those identified in transcriptomic studies using the brains of patients with ASD. The initial dataset used 58 cortex samples, including 29 autistic subjects and 29 controls (Dataset_1), and 235 upregulated and 209 downregulated DEGs were detected [48]. The subsequent dataset encompassed 384 autistic subjects and 341 controls (Dataset_2), identifying 1944 upregulated and 2279 downregulated DEGs [49]. Lastly, the third dataset (Dataset_3), involving 133 autistic subjects and 155 controls, revealing 746 upregulated and 865 downregulated DEGs [50]. Despite being across different species, our analysis revealed a significant portion of the DEGs identified in the *MIR137*^*–/–*^ miniature pig brain displayed similar changes to those observed in the brains of ASD patients (Supplementary Fig. 2 and Supplementary Table 3). To identify the most consistent DEGs, we overlapped the DEGs from three datasets, revealing 62 genes were upregulated and 50 genes were downregulated across all datasets. Notably, approximately 11.29% (7 out of 62 hypergeometric test, P-value = 0.0192) of the upregulated DEGs and 48% (24 out of 50, hypergeometric test, P-value = 8.20e–18) of the downregulated DEGs demonstrated consistent changes between the ASD patient brain and the *MIR137*^*–/–*^ miniature pig brain (Fig. 2G, Supplementary Fig. 2 and Supplementary Table 3).

These findings suggest that the loss of miR-137 in the miniature pig brain leads to the dysregulation of genes associated with neurodevelopment, and cumulatively, these genes may contribute to the pathogenesis of neurodevelopmental disorders.

### Comparative analysis of mouse, miniature pig, and hiPSC-derived neurons reveals distinct transcriptomic features in the brain of *MIR137*^*–/–*^ miniature pig

**Fig. 3:**
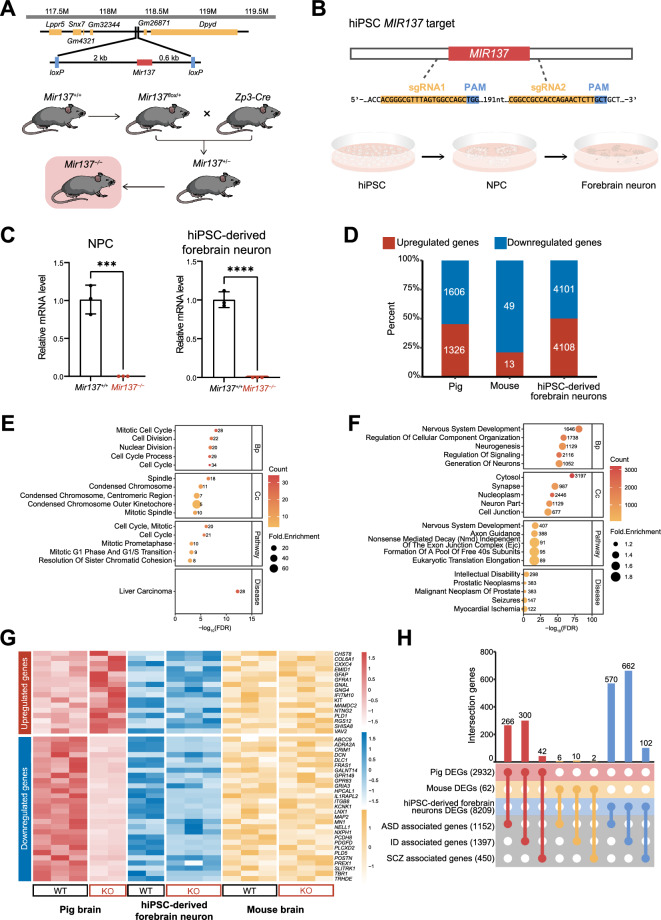
Comparative analyses of miR-137 deficiency in mouse, pig and hiPSC-derived neuron models. **A** Generation of the *Mir137* knockout mice. A targeting vector was designed to disrupt the *Mir137* gene via homologous recombination in mouse embryonic stem cells, with two loxP sites inserted upstream (~ 2 kb) and downstream (~ 0.6 kb) of the *Mir137* gene (top). Crossing with Zp3-Cre mice led to the specific deletion of *Mir137* in the germline, resulting in the generation of homozygous *Mir137* knockout (*Mir137*^*–/–*^) mice (bottom). **B** Generation of *MIR137* knockout hiPSC-derived forebrain neuron. Specific sgRNAs were designed and constructed to target the human *MIR137* gene. The CRISPR/Cas9 gene editing technique was used to deliver Cas9 and sgRNAs into hiPSC via electroporation. Monoclonal cells with CRISPR/Cas9-induced *MIR137* deletion (*MIR137*^*–/–*^) were isolated and utilized to generate neural progenitor cells (NPCs) and forebrain neurons. **C** qPCR verification of decreased expression of mature miR-137 in both *MIR137*^*–/–*^ hiPSC-derived NPCs and forebrain neurons. Data are presented as means ± SEM. Statistical significance was tested with unpaired t-test, with ***p < 0.001 and ****p < 0.0001. **D** Number and percentage of DEGs in pig, mouse and hiPSC-derived forebrain neurons. Mouse and pig gene symbols were converted to human gene symbols using Ensembl Biomart for cross-species comparison. Genes that could not be converted were excluded from the analysis. **E**–**F** Top 5 GO terms, enriched pathways and disease associations for DEGs identified in *Mir137*^*–/–*^ mouse brain (**E**) and *MIR137*^*–/–*^ hiPSC-derived forebrain neurons (**F**). **G** Heatmap showing gene expression of neuronal marker genes across species. Neuronal marker genes overlapping with DEGs identified in *MIR137*^*–/–*^ miniature pig was included. Marker genes were sourced from a public database (http://xteam.xbio.top/CellMarker/). **H** UpSet diagram showing the intersections of DEGs in miniature pig, mouse, hiPSC-derived forebrain neurons, and genes associated with autism spectrum disorder (ASD), intellectual disability (ID), and schizophrenia (SCZ)

To assess whether the miniature pig model can provide a more accurate representation of NDD in humans, we utilized a previously reported mouse model. In the mouse model, complete deletion of *Mir137* gene leads to lethality between postnatal day 14–21 (P14–P20) [17]. Since the *MIR137*^*–/–*^ miniature pig in this study died shortly after birth, we chose to use neonatal P0 mice for comparison in the subsequent experiments (Fig. 3A). Moreover, we established a human induced pluripotent stem cell (hiPSC) line with *MIR137* gene deletion and induced the differentiation of hiPSC into neural progenitor cells (NPC) and forebrain neurons (hiPSC-derived forebrain neuron) (Fig. 3B and Supplementary Fig. 3A). Quantitative PCR (qPCR) analysis confirmed that the expression levels of mature miR-137 were significantly reduced in *MIR137*^*–/–*^ iPSC-derived NPC and forebrain neuron (Fig. 3C).

RNA was then extracted from the cortical tissue of P0 mice and hiPSC-derived forebrain neurons for RNA-seq analysis. In *Mir137*^*–/–*^ mice, we identified a limited number of DEGs, including 19 upregulated DEGs and 51 downregulated DEGs (Fig. 3D, Supplementary Fig. 3B and Supplementary Table 3). In contrast, we observed more than 8,000 gene that significantly changed in the hiPSC-derived forebrain neurons due to miR-137 deletion, with many of these gene alterations consistent with those observed in the *MIR137*^*–/–*^ miniature pigs’ brain (Fig. 3D, Supplementary Fig. 3C and Supplementary Table 3). Through GO, pathway enrichment, and disease-related analyses, we found that the functions of mouse DEGs mainly focused on cell cycle, chromosomal changes, and unrelated to brain diseases but linked to liver carcinoma (Fig. 3E). On the other hand, the functions of DEGs in hiPSC-derived forebrain neurons were primarily associated with neurodevelopment, synaptic function, and axon guidance (Fig. 3F). Particularly, disease-related analysis revealed a significant association between DEGs in hiPSC-derived forebrain neurons and neurodevelopmental disorders, including ID and epilepsy (Fig. 3F), consistent with the results observed in the *MIR137*^*–/–*^ miniature pig brain (Fig. 2F).

Both the DEGs in the *MIR137*^*–/–*^ miniature pig brain and hiPSC-derived forebrain neurons were significantly enriched in neuron development and synapse formation, further supporting the crucial role of miR-137 in neuronal function [17, 51–53]. To further investigate the impact of miR-137 loss on gene expression, we examined the changes in neuronal marker genes across species. In the *MIR137*^*–/–*^ miniature pig brain, we identified a total of 16 upregulated and 28 downregulated neuronal marker genes (Fig. 3G and Supplementary Table 4). Interestingly, these genes exhibited a highly similar change pattern in both the miniature pig brain and hiPSC-derived forebrain neurons. However, minimal gene expression changes were observed in these same genes in the mouse brain, suggesting that our miniature pig model may better reflect the alteration in critical neuronal genes resulting from the loss of miR-137 in human during the neonatal stage.

We then tried to explore the association of the DEGs identified from the miniature pig, mouse, and hiPSC-derived forebrain neurons with NDD. To achieve this, we overlapped these DEGs with the risk genes linked to common neuropsychiatric disorders, including ASD, ID, and schizophrenia (Supplementary Table 4). As shown in the UpSet diagram (Fig. 3H), a significant overlap was observed between the DEGs identified in the *MIR137*^*–/–*^ miniature pig brain and ASD-associated genes (266 out of 1152 genes, hypergeometric test, P-value = 5.34e–42), as well as ID-associated genes (300 out of 1397 genes, hypergeometric test, P-value = 2.97e-41). This suggests that miR-137 may play critical roles in NDD by directly regulating genes involved in these disorders. As expected, similar findings were also noted in the *MIR137*^*–/–*^ hiPSC-derived forebrain neurons, while a limited association was observed between mouse DEGs and disease risk genes (Fig. 3H).

Taken together, these results suggest that the miniature pig model serves as a reliable tool for investigating gene expression changes associated with NDD in humans at molecular level.

### Identification of human-specific miR-137 targets in the ***MIR137***^***–/–***^ miniature pigs

**Fig. 4:**
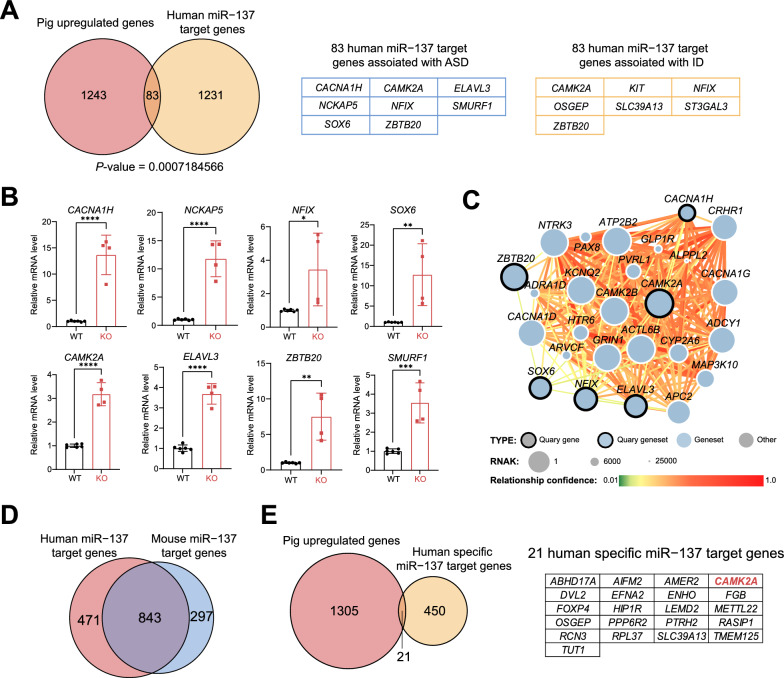
Identification of human-specific miR-137 targets in the *MIR137*^*–/–*^ miniature pigs. **A** The upregulated DEGs in the *MIR137*^*–/–*^ miniature pigs are significantly enriched with human miR-137 target genes (1314 genes obtained from TargetScanHuman 8.0). Among these, eight genes are associated with ASD, and seven genes are associated with ID. **B** qPCR verification of selected human miR-137 target genes in the brain of *MIR137*^*–/–*^ miniature pig. **C** miR-137-mediated regulation in ASD risk genes interactome. A predicted ASD-gene network exploring brain-specific interactions between ASD risk genes was generated, including the selected upregulated miR-137 target genes in the brain of *MIR137*^*–/–*^ miniature pig (dots marked with bold lines). Upregulated miR-137 predicted targets might potentially influence an adjacent ASD candidate gene’s expression, such as *CAMK2B*, *GRIN1* and *CACNA1G* were previously reported as associated with ASD and ID. **D** The 843 genes identified in the study were found to be targeted by both human and mouse miR-137. **E** The 21 DEGs that were upregulated in the *MIR137*^*–/–*^ miniature pig were human-specific miR-137 target genes

In the cytoplasm, mature miRNAs bind to Argonaute proteins to form the RNA-induced silencing complex (RISC) [54]. Through binding to the 3'UTR of target genes in the cytoplasm, miRNAs facilitate mRNA degradation or translation inhabitation, thus exerting negatively regulation on the expression of downstream target genes [54]. Among the 1326 upregulated DEGs (with corresponding human gene symbols) identified in the *MIR137*^*–/–*^ miniature pig brain, we found a significant enrichment of miR-137 targets (83 genes) (Fig. 4A and Supplementary Table 5). Among these genes, we identified 8 genes were reported as ASD risk genes, including *CAMK2A*, *NFIX*, *ZBTB20*, *CACNA1H*, *ELAVL3*, *NCKAP5*, *SMURF1*, and *SOX6*; while 7 genes were associated with ID, such as *CAMK2A*, *NFIX*, *ZBTB20*, *KIT*, *OSGEP*, *SLC39A13*, and *ST3GAL3*. The expression levels of selected miR-137 were validated by qPCR experiments (Fig. 4B). Given the potential role of miR-137 in ASD, we constructed a predicted ASD-gene network to explore brain-specific interactions among ASD risk genes, including the selected upregulated miR-137 target genes (Fig. 4C). In the predicted network, we found that these upregulated miR-137-predicted targets, particularly *CAMK2A* and *CACNA1H*, potentially affected the expression of adjacent genes associated with ASD and ID, including *CAMK2B*, *GRIN1* and *CACNA1G* [55–57].

By comparing the predicted miR-137 targets between humans and mice, we identified 843 genes shared by both species, with 471 human-specific and 297 mouse-specific miR-137 targets (Fig. 4D and Supplementary Table 5). Interestingly, we observed that 21 upregulated DEGs identified in the *MIR137*^*–/–*^ miniature pig brain were human-specific, while no such upregulated miR-137 target genes were found to be human-specific in *Mir137*^*–/–*^ mice brain (Fig. 4E). Notably, we identified *CAMK2A* (Calcium/Calmodulin Dependent Protein Kinase II Alpha) as a human-specific miR-137 target gene in the *MIR137*^*–/–*^ miniature pig brain. *CAMK2A* is a crucial gene that contributes to various aspects of brain function, including learning, memory, synaptic plasticity, and neuronal signaling [58]. Dysregulation of *CAMK2A* has been linked to cognitive impairments and NDD, including ASD and ID [55, 59]. The increased expression of *CAMK2A* in the *MIR137*^*–/–*^ miniature pig brain could potentially lead to cellular, psychological, and behavioral alterations in the miniature pig following the loss of miR-137.

## Discussion

Given the substantial individual variations and the challenges in accessing brain tissue from patients with NDD for scientific research, animal models that closely mimic the clinical features of NDD are essential for investigating the their pathogenesis [60]. While mice and rats are commonly used model animals as primary NDD models in basic research and preclinical trials [61], they do not fully replicate the complex clinical features of NDD due to their evolutionary distance from humans and differences in brain structure, such as the lack of cortical sulcus structure [33]. Although non-human primates can serve as better animal model bridging rodent models of human diseases, their smaller population size, higher costs, and ethical considerations limit their utility in research [33]. In this study, we employed CRISPR/Cas9 in conjunction with SCNT to successfully generate the *MIR137*^*–/–*^ miniature pig by targeting a small DNA fragment (73-bp or 74-bp) within the pig *MIR137* gene. Analysis of brain transcriptomic alterations revealed that gene expression changes in the miniature pig model more closely mirrored those in humans following the loss of miR-137. This positions our miniature pig model as an ideal animal model for NDD, including ASD and ID. To the best of our knowledge, this represents the first gene-edited pig model of NDD, highlighting the significant utility of miniature pig for the study of human neurodevelopment and related disorders.

This study has characterized the transcriptomic features of the *MIR137*^*–/–*^ miniature pig and provides multiple lines of evidence supporting its suitability as an alternative animal model for NDD. Firstly, we observed significant changes in genes associated with neuronal development in the *MIR137*^*–/–*^ miniature pig, mirroring previous findings from transcriptomic analyses using post-mortem human brain tissue samples from patients with ASD [48]. This consistency strongly supports the proposed role of miR-137 in regulating the pathogenesis of NDD. Secondly, the disease association analysis unveiled a significant correlation between the DEGs and ID, indicating potential cognitive issues in the *MIR137*^*–/–*^ miniature pig model. Of particular interest is the association with epilepsy, which has been frequently reported in the patients with NDD [62, 63]. Studies have shown that decreased expression of miR-137 is associated with epileptic phenotypes in brain tissues from patients and mouse models [64, 65]. However, apparent epileptic seizures were not observed in the *Mir137*^*–/–*^ mouse model [17], suggesting that the *MIR137*^*–/–*^ miniature pig model may better illustrate the epileptic symptom seen in patients with NDD. Thirdly, by generating *MIR137*-deficient pig and mouse models and hiPSC-derived forebrain neurons, we conducted a systematic investigation into the altered gene expression resulting from the absence of miR-137 across different species. Notably, the *Mir137*^*–/–*^ mouse model did not show significant changes in gene expression at the fetal stage. While hiPSC-derived forebrain neurons only represent the function of miR-137 in a single, yet crucial, brain cell type, our results indicate that the miniature pig model may more accurately mimic human gene expression patterns compared to the mouse model. Fourthly, in the *MIR137*^*–/–*^ miniature pig model, we have discovered the upregulation of several human-specific miR-137 targets, such as *CAMK2A* that is closely linked to ASD and ID [55, 59]. The pathogenic functions of these crucial genes mediated by miR-137 can be further explored in the current miniature pig model.

Various miniature pig breeds, such as Göttingen miniature pig and Yucatan miniature pig, have been extensively utilized in biomedical research for a wide range of studies, including tumor research, cardiovascular disease, diabetes, and drug safety evaluations [40, 43–46, 66]. The average birth weight of the *Diannan* miniature pig breed, used in our study, is approximately 0.64 kg. By the age of 6 months, these pigs typically reach sexual maturity, with an average weight of around 16.45 kg. Additionally, they have significantly contributed to the development of animal models for human diseases and the production of donor pigs for xenotransplantation [47, 67–69]. Previous studied have shown that patrial loss of miR-137 contributes to the pathogenesis of ASD and ID [17, 29–31, 70]. However, due to the costs associated with generating and breeding mutant miniature pigs, as well as the technical challenges of targeting only a single allele of the *MIR137* gene using CRISPR/Cas9-based gene editing [71], our initial focus was to generate homozygous *MIR137* KO miniature pig. Unfortunately, in this study, we were only able to obtain a limited number of newborn miniature pigs. This outcome was not unexpected as no patient with homozygous loss of *MIR137* genes have been reported with 1p21.3 microdeletion [29–31]. Additionally, our previous study demonstrated that complete loss of miR-137, either in the germline or specific brain cell types, resulted in postnatal lethality in mouse models [17, 32]. Moving forward, further efforts are necessary to generate heterozygous *MIR137* KO miniature pigs and comprehensively assess their performance in behavioral assays related to core clinical symptoms of NDD. In the future investigations, it would be interesting to explore the injection of single sgRNA and Cas9 protein into fertilized ova, accompanied by the inclusion of dCas9 to attenuate the cleavage efficiency of the CRISPR system, thereby aiming to obtain heterozygous *MIR137*^+*/–*^ piglets.

## Conclusions

In summary, our study has shown that the loss of miR-137 can disrupt genes crucial for neurodevelopment and associated disorders in *MIR137*^*–/–*^ miniature pigs, which offers several advantages over mouse models. This establishes a miniature pig model that is well-suited for the investigation of NDD. The insights gained from this miniature pig model shed light on the potential use of miniature pigs in studying human neurodevelopmental disorders.

## Methods

### Animals and ethics statement

All the animal procedures were approved by the Institutional Animal Care and Use Committees (IACUC) of Yunnan University or Yunnan Agricultural University. The *Diannan* miniature pig fibroblast cell line and surrogate sows used in this study were raised at Laboratory Animal Centre of Yunnan Agricultural University. *Mir137*^+*/*+^ and *Mir137*^*–/–*^ mouse strains have been described previously [17, 32]. All mice were kept on a 12-h light/dark cycle (7 a.m.–7 p.m. light period) and 55% humidity in a holding room maintained at a temperature of 22 ± 1 °C with ad libitum access to a standard pellet diet and water. The pups were kept with the dams until weaning at postnatal day 21 (P21). After weaning, mice were divided into groups (maximum of 5 per cage) according to sex.

### Construction and *MIR137* knockout miniature pig

CRISPR/Cas9 gene editing system was used to target *MIR137* gene (Chr4, NC_010446.5) in pigs. Primers were designed for detecting SNPs in the region of *MIR137* gene (Supplementary Table S1). The sgRNA design, transfection and gene editing cell screening procedures were conducted following previously established protocols [72, 73]. In briefly, four sgRNAs were designed and tested in pig iliac artery endothelial cell (PIEC), and sgRNA with high editing efficiency were selected to construct PGL3-U6-sgRNA Puro vector based on the T7 endonuclease I (T7EI) cleavage assay results. The PGL3-U6-sgRNA1-Puro, PGL3-U6-sgRNA2-Puro and spCas9 plasmids (1:1:2) were co transfection into fetal fibroblasts of *Diannan* miniature pig (Supplementary Table S1). Then, the cells were screened for 2 days with a treatment of 2.0 μg/mL of puromycin after 48 h of transfection. The surviving cells were cultured into single-cell colonies by using extreme dilution method. After approximately 12 days, single cell colonies were harvested and genotyped by PCR, T7EI and sequencing. The biallelic *MIR137* knockout cell colonies were selected as the donor cell for somatic cell nuclear transplantation (SCNT). SCNT and embryo transfer were performed as described in our previous studies [74]. *MIR137* knockout fibroblast cells were used as donor cells to inserted into the perivitelline space of an enucleated oocyte. After electrical fusion and activation, the reconstructed embryos were cultured in PZM-3 medium. Then, the reconstructed embryos were transplanted into the oviducts of the recipients, the piglets were born at approximately 114 days of pregnancy.

### Genotyping of ***MIR137***^***–/–***^ miniature pig

The cortical brain tissues of miniature pig were lysed in 600 μL lysis buffer and then digested overnight at 55 °C with 20 μL Protease K (25 mg/ml). 4 μL RNaseA was added and mixed completely, then digested at 37 °C for 15 min. Then the gnomic DNA was extracted with 600 μL Phenol:Chloroform:Isoamyl Alcohol (25:24:1). The primers used in these experiments are shown in Supplementary Table 1.

### Whole-genome sequencing (WGS) analysis

Genomic DNA was extracted from one *MIR137*^*–/–*^ miniature pig and one newborn wild-type control pig. The DNA was used for generate PCR free DNBSEQ library and sequenced by BGISEQ-500 (BGI Company). The clean data (clean reads) were obtained by SOAPnuke. We removed the reads meet any of the following criteria: (1) more than 25% match the adapter sequence; (2) more than 50% bases having a quality value lower than 20; and (3) more than 3% N in the read. All the downstream analyses were based on clean data with high quality. To align the clean reads, we used the Burrows-Wheeler-Alignment Tool (BWA) against the susScr11 reference genome from UCSC. We then assessed the depth and coverage of the aligned reads using samtools and bedtools. Next, we applied the Genome Analysis ToolKit (GATK) for Base Quality Score Recalibration and used HaplotypeCaller to identify insertions or deletions on the porcine genome.

### Generation of *MIR137* knockout hiPSC-derived neurons

Wild-type and *MIR137*^*–/–*^ hiPSC lines were generated and purchased from Cyagen company (China). In brief, appropriate sgRNA were designed and constructed targeting human *MIR137* gene. The CRISPR/Cas9 gene editing technique was employed to deliver Cas9 and sgRNA into hiPSC via electroporation. Monoclonal cells with CRISPR/Cas9-induced *MIR137* deletion were isolated and confirmed by PCR genotyping. The primers used in these experiments are shown in Supplementary Table 1. These wild-type and *MIR137*^*–/–*^ hiPSC lines were utilized to generate neural progenitor cells (NPCs) and forebrain neurons as follows:

*Generation of NPCs.* In brief, Day 0: To begin, coat one well of a tissue culture-treated 6-well plate with Corning Matrigel. Add Y-27632 to STEMdiff^™^ Neural Induction Medium + SMADi (StemCell Technologies, 08581) to achieve a final concentration of 10 µM. Prior to usage, warm all reagents in a water bath at 37°C. Utilize a microscope to visually identify areas of differentiation in the hiPSC culture and eliminate these regions. Rinse the dish once with 5–10 mL of sterile PBS and then aspirate. Introduce 3 mL of Gentle Cell Dissociation Reagent and incubate at 37 °C for 8–10 min. Using a pipettor, gently pipette the cell suspension up and down 3–5 times to detach any remaining attached cells. Transfer the cells into a 15 mL or 50 mL conical tube using a 5 mL serological pipette. Rinse the dish with 10 mL of DMEM/F-12 and combine it with the tube containing the single-cell suspension. Assess the cell viability using Trypan Blue and a hemocytometer. Centrifuge the cells at 300 × g for 5 min. Carefully remove the supernatant and resuspend the cells in STEMdiff^™^ Neural Induction Medium + SMADi + 10 µM Y-27632 to achieve a final concentration of 2 × 10^5^ cells/cm^2^. Add 2 mL of the cell suspension to a single well of the matrix-coated 6-well plate. Place the plate in a 37°C incubator. Day 1–6: Perform a complete medium change every day. Day 7: Passage Cells. Begin by coating one well of a tissue culture-treated 6-well plate with Corning^®^ Matrigel^®^. Warm sufficient volumes of STEMdiff^™^ Neural Induction Medium + SMADi, DMEM/F-12 with 15 mM HEPES, and ACCUTASE^™^ at 37 °C. Remove the medium from the well containing the NPCs and add 1 mL of ACCUTASE^™^. Incubate at 37 °C for 5–10 min. Gently pipette the cell suspension up and down using a 1 mL pipettor to detach any remaining attached cells. Add 5 mL of DMEM/F-12 into the well and transfer the NPC suspension to a 15 mL conical tube. Centrifuge at 300 × g for 5 min. Carefully remove the supernatant and add 1 mL of complete STEMdiff™ Neural Induction Medium + SMADi. Determine the cell viability using Trypan Blue and a hemocytometer. Plate the cells at the desired density of 2 × 10^5^ cells/cm^2^ in 2 mL of STEMdiff™ Neural Induction Medium + SMADi into a single well of the newly matrix-coated 6-well plate. Place the plate in a 37°C incubator. Day 8–13: Perform a complete medium change every day. Day 14: Follow the same procedure as on day 8. Day 15–21: Perform a full medium change daily.

*Generation of forebrain neurons from NPCs.* In brief, Day 22–23: Begin by diluting Poly-L-ornithine hydrobromide (PLO) solution in phosphate-buffered saline (PBS) to achieve a final concentration of 15 µg/mL. Coat the cultureware with the PLO solution to cover the entire growth surface evenly. Incubate at 37 °C and 5% CO2 for 2 h or seal the cultureware and incubate overnight at 2–8 °C. Prepare a working solution of laminin at 5 µg/mL in DMEM/F-12. Rinse the PLO-coated vessel twice with sterile PBS. Remove the PBS and add the laminin solution to cover the entire growth surface. Incubate as previously described. Aspirate the laminin solution just before seeding the cells. Passage the cells as single cells using ACCUTASE^™^. Incubate at 37 °C for 5–10 min. Dislodge any remaining attached cells by gently pipetting the cell suspension up and down using a 1 mL pipettor. Transfer the suspension to a 15 mL conical tube after adding 5 mL of DMEM/F-12. Centrifuge at 300 × g for 5 min. Carefully remove the supernatant and add 1 mL of complete STEMdiff^™^ Neural Induction Medium + SMADi. Count the viable cells using Trypan Blue and a hemocytometer. Plate the cells in a coated well of a 6-well plate at a density of 80–125,000 cells/cm^2^ in 2 mL STEMdiff^™^ Neural Induction Medium + SMADi. Incubate as directed. The following day, replace the medium with 2 mL of STEMdiff^™^ Forebrain Neuron Differentiation Medium (StemCell Technologies, 08600). Incubate as before. Day 24–29: Perform a full medium change daily. Day 30: Passage the cells as single cells using ACCUTASE^™^. Incubate at 37 °C for 5–10 min. Dislodge any remaining attached cells by gently pipetting the cell suspension up and down using a 1 mL pipettor. Transfer the suspension to a 15 mL conical tube after adding 5 mL of DMEM/F-12. Centrifuge at 300 × g for 5 min. Carefully remove the supernatant and add 1 mL of complete STEMdiff^™^ Neural Induction Medium + SMADi. Count the viable cells using Trypan Blue and a hemocytometer. Plate the cells in a PLO/Laminin coated well of a 6-well plate at a density of 6 × 10^4^ cells/cm^2^ in 2 mL STEMdiff^™^ Forebrain Neuron Maturation Medium (StemCell Technologies, 08605). Day 31–44: Perform a full medium change every 2–3 days. Day 45: Collect the cells using 1 mL ACCUTASE^™^ and 20 µL papain for one well of a 6-well plate. Incubate at 37 °C for 15 min. Dislodge any remaining attached cells by gently pipetting the cell suspension up and down using a 1 mL pipettor. Transfer the suspension to a 15 mL conical tube after adding 5 mL of DMEM/F-12. Centrifuge at 300 × g for 5 min. Carefully remove the supernatant and add 1 mL DPBS to the suspended cells. Centrifuge at 300 × g for 5 min. Carefully remove the supernatant and use cells for downstream analysis or store them at −80 °C.

### RNA extraction and real-time qPCR analysis

RNA was extracted from mouse brain, miniature pig brains or hiPSC-derived neurons by using TRIzol (1 mL for 50–100 mg brain tissue or 2–5 × 10^6^ cells), and quantified by Nanodrop. 20 µg of RNA was revised transcribed by Revert Aid First Strand cDNA Synthesis Kit (Thermo Scientific #K1622) or miRNA 1st Strand cDNA Synthesis Kit (by tailing A) (Vazyme #MR201). Quantitative PCR (qPCR) was run with cDNA input in a 20 μL reaction using 2 × SYBR Green PCR Master Mix. For analysis, the *ΔΔCt* method was used to calculate the relative fold gene expression of samples. The housekeeping gene *GAPDH* and U6 served as control for qPCR. The primers used in these experiments are shown in Supplementary Table 1.

### RNA-seq sample processing, library preparation, sequencing, and data analysis

After extracting RNA from mouse brain, miniature pig brains or hiPSC-derived neurons, RNA’s total amounts and integrity were assessed using the RNA Nano 6000 Assay Kit of the Bioanalyzer 2100 system (Agilent Technologies, CA, USA). For library preparation, 1.5 mg of total RNA (RIN R 6.8) was used with VAHTS Universal V8 RNA-seq Library Prep Kit for Illumina (Vazyme, NR605). After the library is qualified, the different libraries are pooled according to the effective concentration and the target amount of data off the machine, then sequenced by the Illumina NovaSeq 6000. The end reading of 150-bp pairing is generated.

The FASTQ format files obtained from the Illumina platform are transformed into short reads (raw data). Sequence quality control is performed using Fastp, which removes reads containing adapters, reads with N bases, and low-quality reads. All downstream analyses are based on clean data of high quality. For alignment, STAR (Version 2.7.9a) aligns the clean reads to the reference genomes: Pig reference: Obtained from Ensembl (Sscrofa11.1); Mouse reference: Obtained from UCSC (GRCm38/mm10); and Human reference: Obtained from UCSC (GRCh38/hg38). Uniquely mapped reads are used for subsequent analyses. RSEM (RNA-Seq by Expectation–Maximization Version 1.3.1) and DESeq2 (v1.42.0) were used to identify differentially expressed genes (DEGs). Genes are considered differentially expressed if the log_2_(FoldChange) is either > 1 or < −1, and the adjusted p-value (p-adjust) is < 0.05. To compare DEGs across species, we converted mouse and pig gene symbols to human gene symbols using Ensembl Biomart. Any genes that cannot be converted are excluded from the analysis.

### Integrated bioinformatics analyses

Gene ontology (GO), pathway and disease-association analyses were performed by Database for Annotation, Visualization and Integrated Discovery (DAVID 2021). The published transcriptomic data of ASD patient brains, used for comparative analysis, are as follows: (1) The initial dataset used 58 cortex samples, including 29 autistic subjects and 29 controls (Dataset_1), and 235 upregulated and 209 downregulated DEGs were detected [48]; (2) The subsequent dataset encompassed 384 autistic subjects and 341 controls (Dataset_2), identifying 1944 upregulated and 2279 downregulated DEGs [49]. (3) Lastly, the third dataset (Dataset_3), involving 133 autistic subjects and 155 controls, revealing 746 upregulated and 865 downregulated DEGs [50]. Predicted ASD-associated gene interaction networks were performed by genome-wide predictions of autism-associated genes (https://asd.princeton.edu/genesets/3) [75]. The disease-associated genes were obtained from research sources as follows: Autism spectrum disorder (ASD) associated genes were obtained from SFARI (https://gene.sfari.org/); Intellectual disability (ID) associated genes were obtained from the Harvard Dataverse (10.7910/DVN/MMUNLR/VMRS7I); Schizophrenia associated genes were obtained from SZDB database [76]. Conserved miR-137 predicted targets in human and mouse were obtained from TargetScan 8.0 [77]. All data visualization was performed using ggplot2 (v3.5.0). Heatmap was generated using the R package pheatmap (v1.0.12). The gene sets overlapped in a Venn diagram using the ggVennDiagram (v1.5.2) package, and the p-values werr calculated using the hypergeometric test by R. UpSet diagram was generated by UpSetR (v1.4.0) and modified using ggplot2 (v3.5.0).

### Statistical analysis

All statistical analyses were performed in Prism 9.0 (GraphPad Software). Datasets were analyzed for significance using unpaired Student’s two-tailed t tests and all data are presented as mean ± SEM. hypergeometric test were performed in R software (http://www.r-project.org/).

### Supplementary Information

Supplementary material 1: Fig. 1. Generation of *MIR137*^*–/–*^ porcine fibroblast cell lines and miniature pigs. (A) Schematic diagram of the *MIR137*-sgRNA targeting sites. (B) T7 endonuclease 1 (T7E1) identification results. (C) Statistical results of grayscale values. (D-E) Genotyping and Sanger sequencing analysis of *MIR137*^*–/–*^ piglets.

Supplementary material 2: Fig. 2. The DEGs identified in ASD patient brain overlapped with the DEGs from *MIR137*^*–/–*^ miniature pig brain. (**A**) Venn diagrams of up-regulated and down-regulated DEGs from three datasets. (**B-D**) Heatmap showing gene expression for the overlapped DEGs identified in the *MIR137*^*–/–*^ miniature pig brain with the DEGs identified in Dataset_1 (B), Dataset_2 (C) and Dataset_3 (D) ASD patient brain.

Supplementary material 3: Fig. 3. Generation and transcriptomic analyses of *Mir137*^*–/–*^ mice and *MIR137*^*–/–*^ hiPSC-derived forebrain neurons. (**A**) Genotyping of *MIR137*^*–/–*^ hiPSC-derived forebrain neurons. (B-C) Differentially expressed genes (DEGs) in *Mir137*^*–/–*^ mice (B) and *MIR137*^*–/–*^ hiPSC-derived forebrain neurons (C). The complete DEGs list is provided in Supplementary Table 2.

Supplementary material 4: Table 1. Sequence information of sgRNAs and primers used in gene editing, genotyping and qPCR.

Supplementary material 5: Table 2. Loss-of-miR-137-induced DEGs in miniature pig brain, mouse brain and hiPSC-derived neurons.

Supplementary material 6: Table 3. Comparison the DEGs identified in the *MIR137*^*–/–*^ miniature pig brain and ASD patient brain.

Supplementary material 7: Table 4. List of ASD, ID and schizophrenia risk genes and neuronal marker genes.

Supplementary material 8: Table 5. Predicted miR-137 target genes in human and mouse.

## Data Availability

The raw sequence data of WGS and RNA-seq reported in this paper have been deposited in the Genome Sequence Archive [78] in National Genomics Data Center [79], China National Center for Bioinformation / Beijing Institute of Genomics, Chinese Academy of Sciences (GSA: CRA015558; and GSA for Human: HRA007017) that are publicly accessible at https://ngdc.cncb.ac.cn/gsa.

## Abbreviations

ADHD: Attention deficit hyperactivity disorder
ASD: Autism spectrum disorders
DEGs: Differentially expressed genes
GO: Gene ontology
GWAS: Genome-wide association studies
hiPSC: Human induced pluripotent stem cell
ID: Intellectual disorders
InDels: Insertions and deletions
miRNAs: MicroRNAs
NDD: Neurodevelopmental disorders
PCA: Principal component analysis
PCR: Polymerase chain reaction
PFF: Porcine fetal fibroblast
RISC: RNA-induced silencing complex
SCNT: Somatic cell nuclear transplantation
WGS: Whole genome sequencing
