# Supplementary figures and images for: Generation and transcriptomic characterization of MIR137 knockout miniature pig model for neurodevelopmental disorders
Source: Cell Biosci. 2024 Jun 28;14:86. doi: 10.1186/s13578-024-01268-8 (PMC11212353; doi:10.1186/s13578-024-01268-8)

Fig. S1

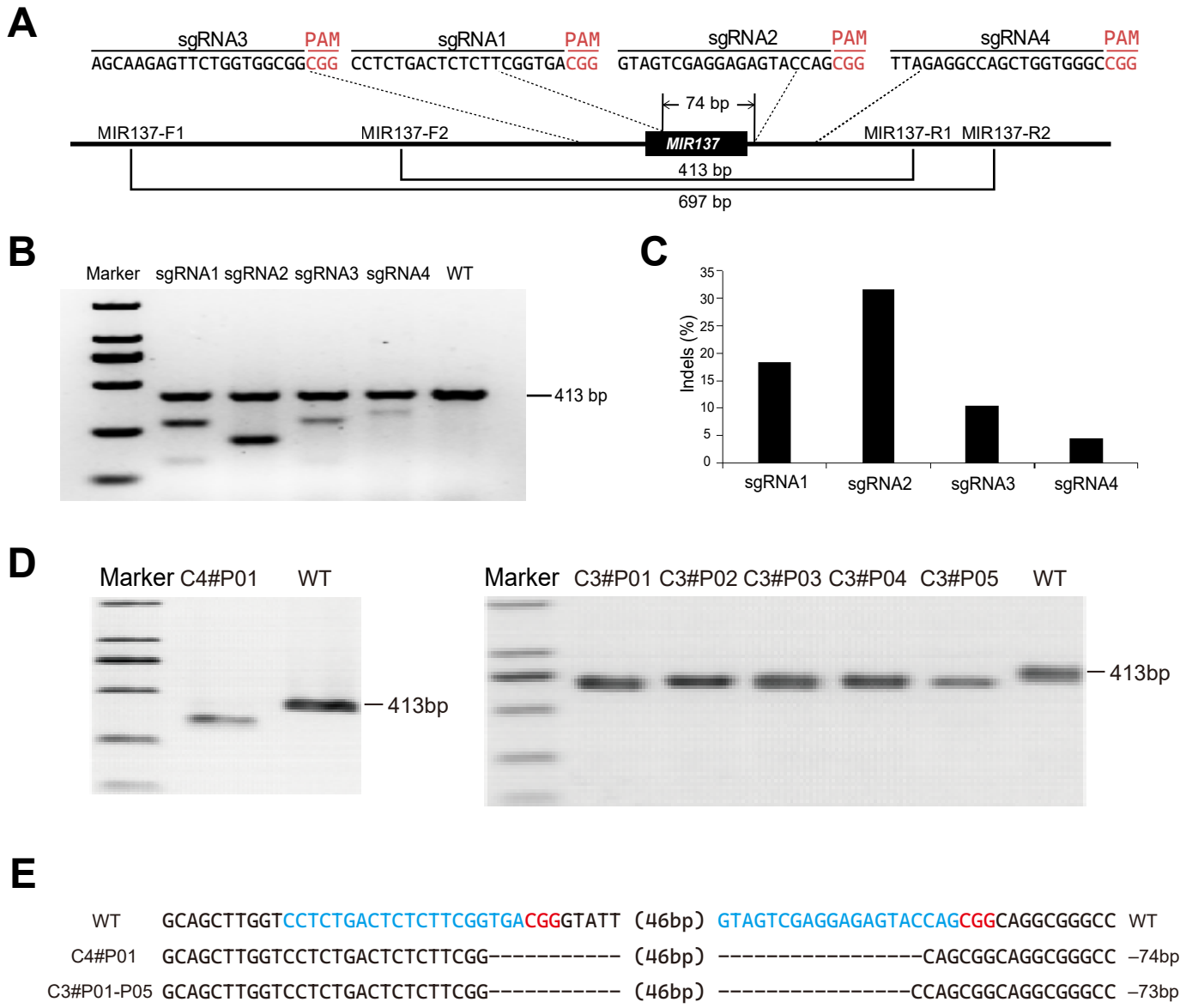

Supplement: Supplementary file 1 — Supplementary material 1: Fig. 1. Generation of MIR137–/– porcine fibroblast cell lines and miniature pigs. (A) Schematic diagram of the MIR137-sgRNA targeting sites. (B) T7 endonuclease 1 (T7E1) identification results. (C) Statistical results of grayscale values. (D-E) Genotyping and Sanger sequencing analysis of MIR137–/– piglets. [file 13578_2024_1268_MOESM1_ESM.pdf]

# Fig. S3

## A

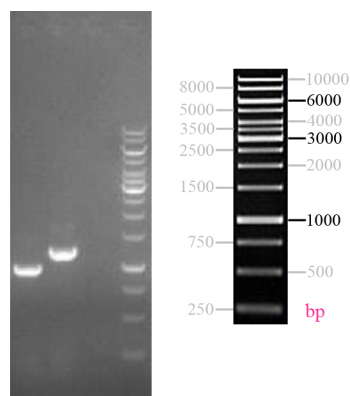

## B

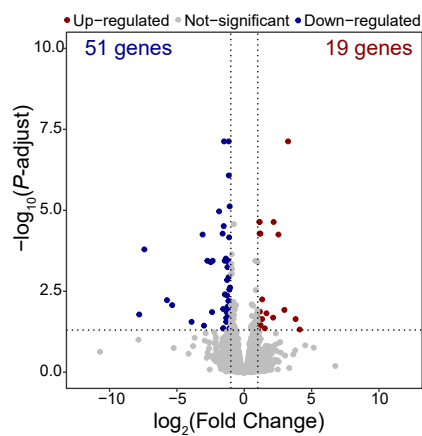

## C

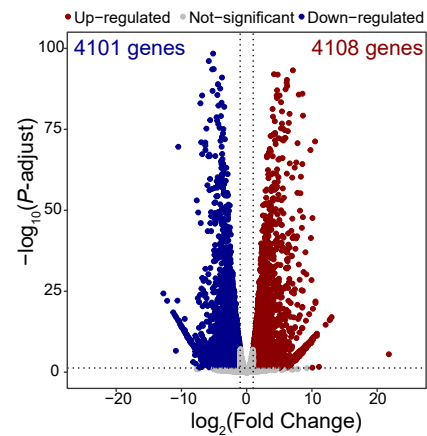

Supplement: Supplementary file 3 — Supplementary material 3: Fig. 3. Generation and transcriptomic analyses of Mir137–/– mice and MIR137–/– hiPSC-derived forebrain neurons. (A) Genotyping of MIR137–/– hiPSC-derived forebrain neurons. (B-C) Differentially expressed genes (DEGs) in Mir137–/– mice (B) and MIR137–/– hiPSC-derived forebrain neurons (C). The complete DEGs list is provided in Supplementary Table 2. [file 13578_2024_1268_MOESM3_ESM.pdf]
