# Supplementary material for: Generation and transcriptomic characterization of MIR137 knockout miniature pig model for neurodevelopmental disorders
Source: Cell Biosci. 2024 Jun 28;14:86. doi: 10.1186/s13578-024-01268-8 (PMC11212353; doi:10.1186/s13578-024-01268-8)

Fig. S2

A

ASD human brain data	DEGs	Gene number	Overlap with pig data	Percentage	P-value
Dataset_1	up	235	26	11.06%	1.75E-05
	down	209	54	25.84%	1.30E-22
Dataset_2	up	1944	145	7.46%	3.46E-10
	down	2279	361	15.84%	9.08E-84
Dataset_3	up	746	54	7.24%	2.90E-04
	down	865	146	16.88%	8.90E-36
3 Datasets overlap	up	62	7	11.29%	0.0192
	down	50	24	48.00%	8.20E-18

B

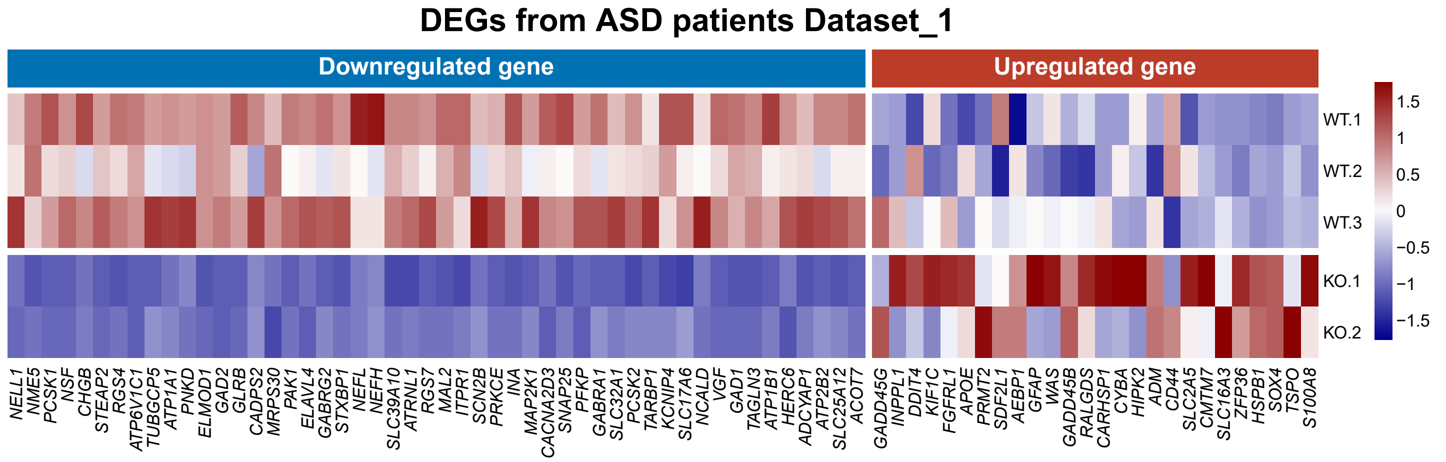

C

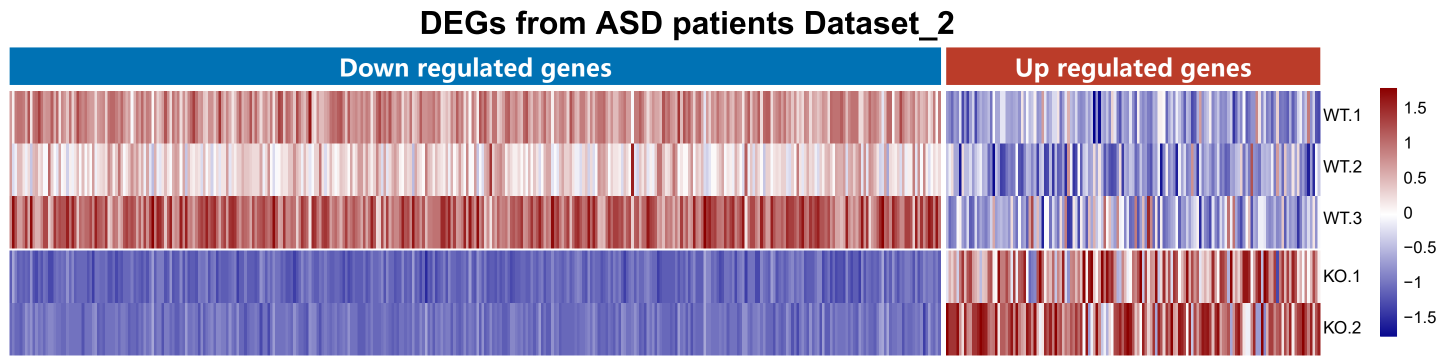

D

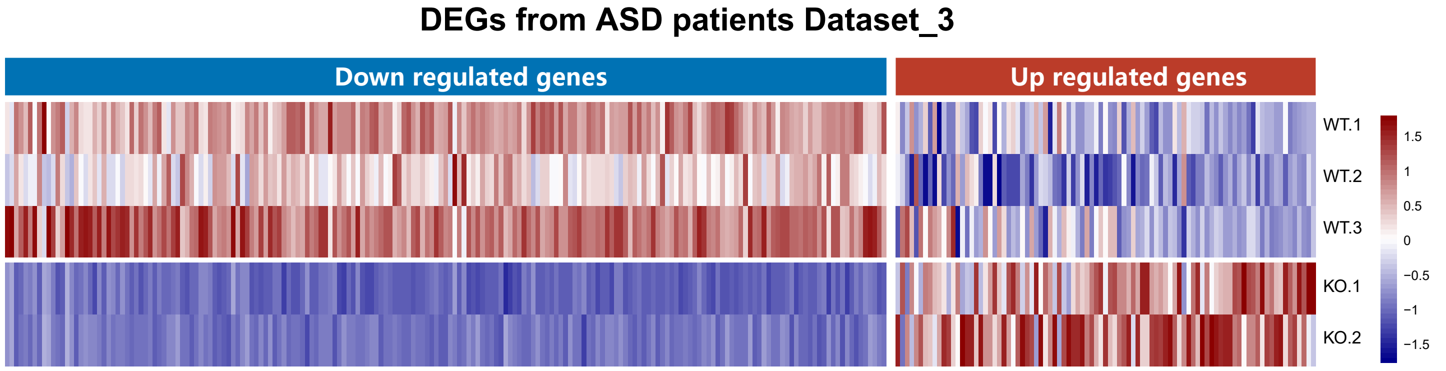

Supplement: Supplementary file 2 — Supplementary material 2: Fig. 2. The DEGs identified in ASD patient brain overlapped with the DEGs from MIR137–/– miniature pig brain. (A) Venn diagrams of up-regulated and down-regulated DEGs from three datasets. (B-D) Heatmap showing gene expression for the overlapped DEGs identified in the MIR137–/– miniature pig brain with the DEGs identified in Dataset_1 (B), Dataset_2 (C) and Dataset_3 (D) ASD patient brain. [file 13578_2024_1268_MOESM2_ESM.pdf]
